# Understanding experiences of cognitive decline and cognitive assessment from the perspectives of people with glioma and their caregivers: A qualitative interview study

**DOI:** 10.1016/j.ijnsa.2024.100179

**Published:** 2024-01-17

**Authors:** Melissa A. Carlson, Elizabeth A. Fradgley, Christine L. Paul

**Affiliations:** College of Health, Medicine, and Wellbeing, University of Newcastle, Level 3, Education Block, John Hunter Hospital University Way, Callaghan, NSW 2308, Australia

**Keywords:** Glioma, Cognitive assessment, Supportive care, Cognition, Cancer

## Abstract

**Background:**

Despite the impact of cognitive decline during brain cancer care, implementing routine cognitive assessment can be challenging. Effective implementation of cognitive assessment necessitates an understanding of implementation from the patient perspective. However, little is known about how people with glioma and their caregivers experience cognitive changes, assessment and support.

**Objective:**

To understand the lived experiences of changes in cognition for people with glioma and their caregivers including experiences of: i) perceived or objectively measured cognitive decline (or absence of decline); ii) cognitive assessment following diagnosis, and; iii) met and unmet cognition-related supportive care needs.

**Design:**

Semi-structured qualitative telephone interviews were conducted with people with gliomas and support persons and analysed using reflexive thematic analysis.

**Setting(s):**

Two Australian cancer services

**Participants:**

18 people with glioma and caregivers

**Methods:**

Semi-structured qualitative telephone interviews were conducted with people with gliomas and caregivers and analysed using reflexive thematic analysis.

**Results:**

People with glioma (*n* = 5) and caregivers (*n* = 13) completed interviews. Four themes were identified: Cognition needs to be considered within the context of glioma diagnosis and treatment; concerns about cognition were initially subordinate to survival but become important; there are challenges identifying and communicating about people with gliomas’ changes in cognition; cognition-related supportive care can be helpful but challenging for people with glioma and caregivers to identify and access.

**Conclusions:**

Changes to cognition can have considerable impacts of people with glioma and their caregivers which may be overshadowed by treatment and survival. A multi-disciplinary approach to timely cognitive screening, structured referral pathways, and communication with caregivers may provide opportunities for support.

**Registration:**

n/a

**Tweetable abstract:**

Identifying cognitive changes in people with glioma is important and challenging. A multidisciplinary approach and inclusion of care coordination and caregivers can help.

**What is already known**

• Cognitive impairment can negatively impact physical and mental health, and quality of life of people with glioma and their caregivers.
• Despite the clinical and prognostic value of routine cognitive assessment with people with glioma, implementation can be challenging.

Alt-text: Unlabelled box

**What this paper adds**

• Barriers to ensuring that healthcare professionals are aware of changes in patient cognition can include inconsistent cognitive assessment, lack of patient cognitive insight, and caregiver discomfort in discussing changes in cognition.
• Opportunities to improve the identification of cognitive changes include the inclusion of neuropsychologists in Multi-Disciplinary Teams, providing direct lines of communication between caregivers and healthcare professionals, and supplementing objective cognitive assessments with subjective cognitive assessments and discussion of symptom intrusiveness.
• Care Coordinators or Social Workers may be ideally placed to support patients and caregivers to navigate accessing support for cognitive changes; A process which may be aided by the development of structured referral pathways.

Alt-text: Unlabelled box

## 1 Background

Primary brain tumours are life-threatening and debilitating conditions with no known lifestyle risk factors, low survival rates and high incidence of chronic disability among survivors (Hahn et al., 2003, Khodamoradi et al., 2017, Leece et al., 2017). World Health Organisation (WHO) low grade (Grade I or II) gliomas have an average median survival of seven years (Affronti et al., 2018, Flores et al., 2014, Armstrong et al., 2016), while high grade (Grade III or IV) tumours have a median survival of one to three years, and a five year survival rate of 10 % (Armstrong et al., 2016, Bayen et al., 2017).

Given the relatively low survival rates for gliomas, optimising health-related quality of life is often a treatment priority for this group (Cheng et al., 2009). People with brain tumours, such as glioma, frequently have cognitive deficits present at diagnosis due to the tumour and these complications may be compounded by multimodal treatment (Kirkman et al., 2022, Parsons and Dietrich, 2021). Cognitive deficits in people with brain tumours may have multi-faceted impacts on their personalities, relationships, leisure, employment, decision making, emotional wellbeing, physical health, quality of life, and survival (Parsons and Dietrich, 2021, Gosselt et al., 2020, Chieffo et al., 2023, Schagen et al., 2014, Lucchiari et al., 2015, Olson et al., 2016). Cognitive deficits can also impact people who are supporting someone with a glioma (Boele et al., 2013, Chen et al., 2021). Pharmaceutical, behavioural, mindfulness, and exercise interventions have shown promise with improving cognitive functioning or managing the impacts of cognitive deficits; but rely on appropriate identification of those who are at risk for or are experiencing cognitive decline (Parsons and Dietrich, 2021, Chieffo et al., 2023, van Lonkhuizen et al., 2019). Available measures for cognitive assessment include objective measures such as neuropsychological batteries and cognitive screens which may identify objective cognitive deficits, or cognitive screens. Subjective cognitive assessment measures include Patient Reported Outcome Measures (PROMs), which indicate self-perceived changes in cognition and related challenges (Olson et al., 2016). Given perceived impairment has been associated with patient distress, poorer quality of life, and it impacts on employment, social functioning, and community integration; it has been suggested that assessing subjective cognition is just as important as assessing objective cognition (Olson et al., 2016, Lycke et al., 2019, Hutchinson et al., 2012).

Implementation of routine assessment to detect cognitive changes and trigger referrals for interventions remains challenging (Olson et al., 2016). Relatively little is known about how people with glioma and their caregivers experience cognitive changes, cognitive assessment, and associated support. A 2022 systematic review found limited data on the acceptability of cognitive assessment among people with glioma (Carlson et al., 2022). A 2020 cross-sectional study by Wong et al. involving older adults (50 years or older) from the general population, reported 92 % of participants had at least one worry about having their cognition assessed or returning concerning cognitive results (Wong et al., 2020). People with glioma are frequently diagnosed at a younger age than other cancer groups and are therefore, likely to have caring and/or employment responsibilities. Consequently, fears about the results of cognitive tests may influence the willingness of people with glioma to complete cognitive assessment (Chieffo et al., 2023, Claus et al., 2015).

The implementation of a routine cognitive assessment in healthcare settings is more likely to be successful if the barriers and facilitators to the practice are understood from the consumer (patient and caregiver) perspective (Grimshaw et al., 2012). Furthermore, principles of person-centred cares require input from consumers to ensure treatment and support options are of value to consumers (Oliver and Bulbeck, 2019). Given the complexity of these topics, an in-depth qualitative approach to their study is warranted.

The aim of this study is to understand the lived experience of changes in cognition for people with glioma and their caregivers including experiences of:

1. Cognition, including observations and feelings about perceived or objectively measured cognitive decline (or absence thereof).
2. Cognitive assessment following the diagnosis.
3. Met and unmet cognition-related supportive care needs.

## 2 Methods

### 2.1 Design

Cross-sectional semi-structured qualitative telephone interviews were completed and analysed using reflexive thematic analysis (Braun and Clarke, 2020). The project was approved by Hunter New England Human Ethics Committee (2019/ETH11694).

### 2.2 Study setting and participants

Eligible participants were: eighteen years of age or older; had received a glioma diagnosis (herein referred to as people with glioma), or were supporting someone with a confirmed glioma diagnosis (herein referred to as caregivers); participated in a pilot study of the Audio Recorded Cognitive Screen (Schofield et al., 2010) and attended at least two appointments within a six-month time frame at one of the study sites. This timeframe ensured there was opportunity for both cognitive assessment and discussion of supportive care. Caregivers who had completed an online survey as part of a pilot study and indicated they could be contacted about further research were first invited to participate in this study; then the person they were caring for was invited to participate. This approach was chosen to be sensitive to the challenges of treatment and recovery for people with glioma. All participants were given time to review an information statement outlining the reason for conducting the research prior to consent.

### 2.3 Procedure and measurement

Telephone interviews were conducted between 13th September 2021 and 8th March 2022 by a PhD Candidate (MC) with qualifications and skills in social science methods and experience in qualitative research with people with a cancer diagnosis and their caregivers. The interviewer (MC) is currently conducting PhD research exploring cognition with people with glioma and their caregivers and has had previous and ongoing contact with participants through another study piloting cognitive assessments (Carlson et al., 2022). A semi-structured interview guide (See Supplementary File 2) was developed exploring: experiences, observations, and feelings about perceived or objectively measured changes in thinking and memory (or absence thereof); experiences of thinking and memory assessment, and; experiences of met and unmet supportive care needs related to changes in thinking and memory

The interviews were completed between two and 17 months after diagnosis, and were between 17 and 56 minutes in duration. The interviews were audio-recorded on a tablet, transcribed by a professional transcription service, de-identified and imported into NVivo 12 software. Reflexive field notes were used to record team observations and reflections. Authors (MC, CP, EF) met throughout recruitment and data collection to reflect on the interviews and field notes. During this process it was agreed that the achieved sample of participants provided rich breadth and depth for analysis (as favoured over data saturation in thematic analysis (Braun and Clarke, 2020)). A subset of these data regarding participants’ experiences of specific aspects of the Audio Recorded Cognitive Screen were analysed separately and reported elsewhere (Carlson et al., 2023). Further detail on the procedure is provided in Supplementary File 3.

### 2.4 Analysis

**Fig. 1:**
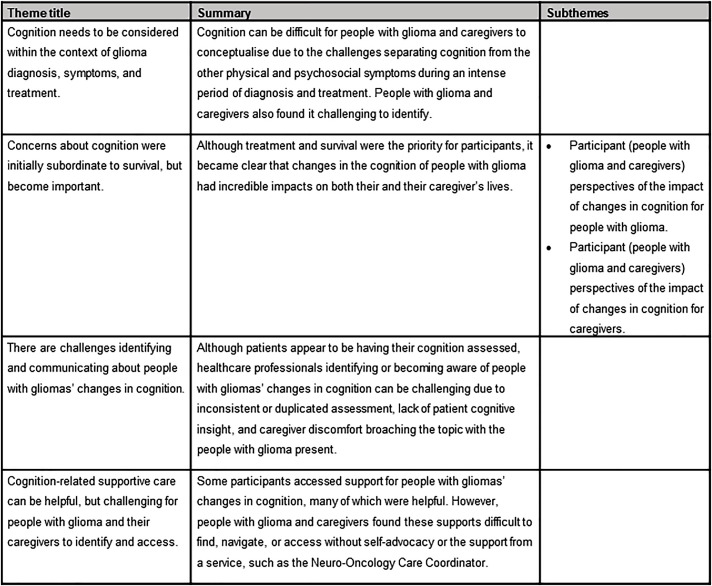
Thematic structure.

Reflexive thematic analysis (Braun and Clarke, 2020) was chosen to allow an in-depth, inductive and interpretive approach which can be used to identify patterns within the data, and is theoretically flexible (Braun and Clarke, 2020). This theoretical flexibility allowed the research to be underpinned by critical realism, which can both centre participants’ voices while also situating those voices within the wider context of health systems and practices (Braun and Clarke, 2020, Brunson et al., 2023). The transcripts were coded by one researcher (MC) in a collaborative and iterative process with three members of the research team (MC, CP, EF). This process is described in detail in Supplementary File 3. The thematic structure is provided in Fig. 1.

## 3 Results

### 3.1 Participants

Twenty-three caregivers indicated an interest in being contacted about the study. Of the 23 interested caregivers, 13 caregivers completed an interview, nine were lost to follow up (contacted the maximum number of times approved by the Human Research Ethics Committee), and one declined to participate. People invited to the study were not required to give a reason for declining to participate. Of the 13 caregivers who completed interviews, eight consented to the research team contacting the people with glioma. Of those, five people with glioma completed interviews, two were lost to follow up, and one declined. Of the 18 participants who completed interviews, five were people with glioma and 13 were caregivers supporting either a partner/spouse (*n* = 10) or parent (*n* = 3) (see Supplementary File 1 for demographic characteristics). Herein, ‘people with glioma’ or ‘caregivers’ will be used to present data from either cohort. ‘Participants’ will be used when the data reflect perspectives of both people with glioma and caregivers.

### 3.2 Qualitative themes

Four themes were identified: (1) Cognition needs to be considered within the context of glioma diagnosis and treatment; (2) Concerns about cognition were initially subordinate to survival, but become important; (3) There were challenges identifying and communicating about changes in cognition, and; (4) Cognition-related supportive care can be helpful, but challenging to identify and access.

#### 3.2.1 Cognition needs to be considered within the context of glioma diagnosis and treatment

During the interviews, it was challenging to discuss cognition as a concept distinct from the overall experience of diagnosis and other physical and psychosocial symptoms related to the tumour and treatment. For participants, receiving a brain cancer diagnosis and treatment plan was a period of ‘just bewilderment, devastation, grief, just a reality, a shock, just an absolute shock’ (Lisa, caregiver). Presenting with symptoms plunged participants into a ‘world-shattering’ whirlwind with near immediate surgery, diagnosis, and intensive treatment.

Throughout the interviews, the interviewer referred to cognition as thinking and memory. However, people used varied language to describe the changes to cognition they experienced or observed. Participants used language such as ‘bit of a blur’, ‘concentration’, ‘disinhibition’, ‘overwhelm’, ‘erratic behaviour’, change in ‘mannerisms’, change in ‘motivation’, ‘forgetfulness’, ‘focus’, being ‘slow’, ‘troubles with problem solving’, ‘understanding’, ‘reasoning’, ‘speech difficulties’, being ‘wonky’ or ‘woozy’, or ‘getting doddery’.

This suggests that there are symptoms of cognitive impairment that participants perhaps found difficult to define or did not conceptualise as falling under the umbrella of cognition.

#### 3.2.2 Concerns about cognition were initially subordinate to survival, but become important

When queried about cognitive concerns, many participants suggested that ‘fighting this disease is the biggest thing’ (Pamela, caregiver). Many described how “there is so much happening so quickly and life changes so drastically, in a moment, that you're just trying to keep up with everything” (James, caregiver). For participants, cognition was secondary to the imminent need to focus on the implications of receiving a potentially life-threatening diagnosis.

“I was thinking more about my kids and dying, basically, rather than thinking about my memory or thinking.” (Lisa, caregiver)

Participants expressed the needed to prioritise coping with the diagnosis, treatment, and more physical symptoms and treatment side effects, before considering the implications of the tumour and treatment on their cognition.

“…just getting through the surgery and then getting the treatment happening afterwards. Yes, the thinking and all of that wasn't a concern…I think physically is more the worry…what we're focused on are just the chemo and the radiotherapy and getting all these scans done and seeing that it's still reduced the swelling. So that's more of a priority for us.” (Carol, caregiver)

Initially, when asked about changes to cognition, some participants stated that they had not observed many changes to cognition and referred to less complex cognitive operations to illustrate this.

“He still knows lots of things, he hasn't lost a lot I don't think … He knows his birthday. He knows where he lives. He …still answers his mobile phone, he still texts, he still does all of those things.” (Donna, caregiver)

However, further discussion of changes in thinking and memory elicited descriptions of subtle or even pronounced changes. As Donna (caregiver) went on to describe ‘But it's probably just little things. He might go to do something and he's forgotten to do it.’ Many participants further described the challenges in changes to memory or capacity to cope with day-to-day life, particularly when it came to more complex cognitive tasks or over-stimulating environments.

“The only thing I have noticed though is she does tend to get stressed if there is too much happening at once.” (Richard, caregiver)

Participants described changes in mood, personality, or the capacity to regulate emotions.

“I noticed with her brain, probably in her last seven or eight months, it was failing. She would forget things. And, thinking of general conversation, was opposite to what she really was as a person. She wasn't abusive … just making silly statements, which was awful for her. It was just awful. So, it was terrible to see her declining like that... And you ask any of her friends, any of our relations, she hasn't got a nasty bone in her body. And just to see the changes she was going through. And she got a bit snappy with me a few times. And I 100 % understood where that was coming from, because it wasn't her.” (Tom, caregiver)

### 3.3 Participant (people with glioma and caregiver) perspectives of the impact of changes in cognition for people with glioma

Although changes in cognition had manageable impacts for some people with glioma such as feeling foggy or forgetful, for others, these changes were more substantial. Participants reported that people with glioma experienced difficulties with daily living such as shopping for groceries, cooking for themselves, managing their finances and administration, managing their medications, and many were no longer able to drive. This meant a loss of independence for those who could no longer independently complete daily living tasks, go to work, or socialise without relying on a caregiver. One caregiver recounted how, upon diagnosis, she discovered that her mother had been struggling with these activities for some time.

“By the time she was diagnosed, when I came here, we found the house in severe state of disarray - squalor - that's the word. And going through I was finding overdue bills back to January …. She had locked herself out of just about every online account she had, emails, the whole bit, just from repeatedly trying wrong passwords.” (Sarah, caregiver)

Participants described how, due to changes in cognition, some people with glioma could become overwhelmed if they were overstimulated in noisy or busy environments. This meant changes to their social lives as well as their relationships with family and friends. Some people with glioma exhibited changes in the way they were able to communicate: For example, becoming less patient than they had been in the past, or quicker to anger with their partners or children when overwhelmed. One caregiver described how his partner was no longer able to handle interruptions from her children as patiently as she had in the past.

“For example, she might be on the computer doing something and then [our son] will come out with a question about school or something and then I will try and help. Then it starts. I think she is trying to do three things at once… I just feel she would have dealt with that differently previously. She wouldn't have had such a short fuse in those situations.” (Richard, caregiver).

Other people with glioma limited their socialising. One caregiver described how his partner who had always been ‘a social butterfly’ was now ‘very much into her shell’ since her diagnosis (Mary, caregiver). Another caregiver described how being overwhelmed by movement and noise meant the person with glioma would limit their social interactions.

“…when the noise ramps up, people have a few drinks or whatever… he just can't be in that noise for too long, whereas before all this, that wouldn't affect him at all.” (Pamela, caregiver)

People with gliomas also worried about how changes in cognition would impact their ability to return to work or caring for their children.

“One of the hardest things was when he went back to work and one day he came home so upset because he said, I don't know if I can do my job anymore. And for someone as stubborn as he is to admit that… it's heart breaking to watch.” (Jess, caregiver)

Some people with glioma were able to return to work, and continue working, though with some trepidation and adjustments. For some, this could mean a slow return to work, with some supervision to ensure they have the cognitive capacity to handle their work. Others adjusted their expectations or work habits. For example, one person with glioma became aware they were no longer able to focus for extended periods. Others implemented strategies to manage difficulties with their memory, such as categorising things in their mind or utilising written notes.

“Short-term memory… there might be times where I have to measure that thing four times for that measurement to stick in my head long enough. And I've found a way of adapting to that now is I'll always have a Texta or a piece of chalk with me and write the measurement down…it does make the guys at work laugh a little bit when they see me measuring the same thing three or four times. But it does get quite frustrating.” (Matthew, person with glioma)

For some people with glioma, the changes in their cognition meant that it was unlikely that they would ever be able to return to work.

“It's possibly impacted me because I'm not quite as… My sister always said I was quite bright… and to do what I was doing I had to be. And then this has happened, and now it's like… I can't probably focus for the whole eight hours, I'm not sure.” Linda, person with glioma)

### 3.4 Participant (people with glioma and caregivers) perspectives of the impact of changes in cognition for caregivers

The changes that people with glioma experienced to their cognition often resulted in changes to the lives and relationships of caregivers and people with glioma. Even subtle changes to cognitive capacity, mood, and personality could require support from the caregiver. For example, forgetfulness meant some people with glioma would need many reminders to take medication or attend appointments. Others needed encouragement to exercise or engage in occupational therapy due to a lack of motivation. One caregiver described how, even though specific action was not often needed, the potential for distress and even seizures meant the person with glioma needed constant supervision.

“Because 90 % of the time, I don't think she needs the help. But then, that 10 % is so critical… You need to be there, and she needs someone to be there to help her, but the person that's there isn't really doing anything at all, but they have to be there and that is just so frustrating…But you'll hear her saying, [James], I need help and you'll walk in, and you'll look at her and you see her staring at you with almost violent eyes like, I need help and gritting her teeth, and you're like this is the first step towards a seizure kind of situation. Because whatever it is that she's taken on too much of or that she's worrying about, thinking too much about, it's just exceeded her capacity to work it out, to cope, to organise. She gets stuck.” (James, caregiver)

Changes, both subtle and pronounced, could change the nature of the person with glioma and caregiver's relationship. For example, one caregiver found that cognitive changes, including a loss of memory and motivation, meant they were regularly pushing the person with glioma to engage with their physiotherapy plan.

“I've become the carer, and the relationship has changed… Yes, I'm the carer, I'm the chauffeur. It's also, in some ways the relationship…. We're at loggerheads with the exercise, that gets pretty tough at times, I just can't cope, but apart from that, there's also my life is more with his now as against we were quite independent.” (Kimberly, caregiver)

Caregivers were also aware of the high risk of the cancer returning, and consequently were constantly monitoring the person they were supporting for the subtle changes in cognition such as memory, movement, mannerisms, speech, moods or stress and anxiety that might be a subtle sign that the cancer has returned.

“I don't think [she's] aware of that, but I'm fully aware of what it was and now I'm looking for it all the time… because one thing they did say to us is, when this comes back, and they said it probably will… just keep a lookout for signs. To me, any change in their mannerisms or their speech or, as in [her] case, their memory, just keep a lookout for those things and, if anything's off at all…” (Ken, caregiver)

Caregivers described the constant vigilance in watching out for and analysing changes.

“… you overthink everything, every little thing that he would say I would monitor it and check it and watch for any changes... It adds that extra level of stress, maybe that's just for me, but you do, you over-think everything, you watch everything.” (Jess, caregiver)

These changes had a considerable impact on the mental and physical lives of caregivers and high levels of need for care of the people with glioma could be the impetus for substantial life changes for the caregiver including moving home or retiring from employment. One caregiver described struggling to manage the additional pressure and either reduced the amount they were working or were no longer able to work, despite help from family and friends.

“I was just shattered… It affected me terribly. And I was still trying to go to work. And then … I couldn't do my job. I couldn't look after her and look after the house. And I was getting some amazing help from my son, my daughter, and my son-in-law. And I couldn't go on anymore… mentally it was affecting me.” (Tom, caregiver)

#### 3.4.1 There are challenges identifying and communicating about people with gliomas’ changes in cognition

All people with glioma in this study had been administered a cognitive assessment as part of a pilot study. However, participants were also asked about any other encounters they had where cognition had been either informally or formally assessed. Participants reported a variety of experiences around cognitive assessment. Some participants could not recall any formal cognitive assessments being carried out. Others reported that they experienced informal assessments or had discussions with a healthcare professional about cognition. Some participants also suspected that they/the person with glioma may have been informally probed about the patients cognition.

“So maybe on some level, yes, I was being tested as to what I recalled from previous consultations and things like that… I was quite upfront with most of the things going on. Yes, possibly on some level I was covertly being tested…” (Matthew, person with glioma)

Some caregivers also reported a learning effect where some common cognitive screens were being used regularly with the person with glioma.

“Oh, I remember the three words. It was the same test. By the time the third person asked him to count backwards by sevens, he was doing it pretty well.” (Kimberley, caregiver)

Some people with glioma lacked cognitive insight into their own changes in cognition. One person with glioma described how she had not been cognisant of her own symptoms when her family took her to the hospital due to confusion and forgetfulness.

“…I just didn't really register that it was happening. They were saying something was wrong, and I went to the hospital, and they asked questions, do you know why you're here? No. But obviously [my partner] had already put down that I was forgetting things or got confused.” (Linda, person with glioma)

Some caregivers described a disparity between the person with glioma and the family's perspective of the person with glioma's cognition. People with glioma would communicate to healthcare professionals that they had not experienced any changes or difficulties in cognition, despite caregivers observing otherwise. Caregivers were reticent to challenge the person they were supporting's perception of their own cognition. Caregivers often avoided these conversations out of worry they might embarrass or anger the person with glioma, or because they did not want to worry or confront them with their concerns.

“They're very standard questions. How are you feeling, has there been any change? That's confronting, because I'm always with [Geoff] and he'll say I'm fine, I feel okay, and I have to give the detail. And if you try and tell the doctor that, oh, look, he's really confused or his confusion's getting worse or anything like that, that's hard, that's confronting, because [Geoff] doesn't realise that.” (Kimberley, caregiver)

One caregiver described how, while her mother was aware of the difficulties she was having with her cognition, her mother did not want to discuss these with the doctor as she was embarrassed but was happy for the caregiver to speak directly with the Neuro–Oncology Care Coordinator about these concerns.

“Well, it's embarrassing. She had a lot of shame about it…it would have made Mum feel terrible, just this long list of, in her mind, all the ways that she'd failed, all the things that she'd done wrong. It's not like that. It's not you, Mum, this is a tumour.” (Sarah, caregiver)

One caregiver also described being unclear what symptoms they should be discussing with their oncologist, stating that some of the cognitive changes fell into a ‘grey area’ between the physical and the psychosocial, suggesting perceptions that psychosocial matters were not the realm of their oncologist.

“…it's a grey area between telling the oncologist what's medical and what's psychological, if you know what I mean, what's therapy. Maybe it's me just not wanting to confront [Matthew] with my thoughts, I don't know, but if they really wanted to know, probably an interview with the carer, they'd get more out of it.” (Kimberley, caregiver)

Caregivers suggested that one way to address this challenge could be by ensuring there are opportunities for caregivers to have direct and private opportunities to discuss their concerns with healthcare professionals. Some caregivers in this study were able to do this via their Neuro–Oncology Care Coordinator who they could call or email directly with concerns without having to confront or worry the person with glioma.

“Because no one ever wants to ring a specialist or email them… But we have this brain care nurse. There was a time when I said, look, heads up, I can't answer these questions in front in the room, I'm going to give them to you by email, a couple of concerns I had. And she would then tell the doctor. That was convenient. But it's like that. You can't – we don't really get nitty-gritty in the regular.” (Kimberley, caregiver)

#### 3.4.2 Cognition-related supportive care can be helpful, but is challenging for people with glioma and caregivers to identify and access

Although some participants suggested support for thinking and memory was not needed, many took steps to either reduce the cognitive side effects of the tumour or treatment, or to manage cognitive changes. Approaches to reducing cognitive side effects included purchasing puzzles and thinking games with the aim of keeping the mind active.

Participants also accessed a variety of supports to help manage changes in cognition including: government financial support in the form of disability and carer's pensions; assistance with daily living (including Aged Care and the National Disability and Insurance Scheme (NDIS), such as support workers to assist with shopping, meal preparation, driving and personal care; physiotherapy to help with motivation for exercising; psychology for help with strategies for remembering, preventing and dealing with overwhelm, and overall support for coping; rehabilitation; speech therapy for help recovering speech as well as strategies for remembering, and; palliative care, which included a 24 hour support line and coordination of home care staff.

Although participants reported that most of the cognition-related supportive care services they accessed were helpful, they encountered challenges with timely discovery and access to these services. Participants reported that they were often unsure of what services were available to them and when and how to access them.

“I guess in some ways we don't even know what questions to ask. And it's very unpredictable. My sister is very frustrated. How do we know when we're going to need this care, or how many months it might be, or are we doing all this and he won't even get to enjoy it, or can we book something six months in advance, that kind of stuff. But I also feel like there's nobody that can tell us that, there is no way of knowing.” (Angela, caregiver)

One person with glioma described accessing rehabilitation and psychological services which he found very helpful for managing his changes in memory at work. However, he felt he needed to advocate for himself to find out what services were available.

“I was a little bit pushy on asking questions on how I can get back on my feet, the quickest way, you know? As soon as I asked them questions, people were more than helpful in guiding me through it. But certainly it wasn't said, this is good for you to do this, or whatever, it was more questions came from me that prompted it…” (Chris, person with glioma)

He expressed concerns that others who are less comfortable communicating their needs to healthcare professionals might be missing out.

Participants also found having someone to advocate for them or to help them to navigate the system, such as a Neuro–Oncology Care Coordinator or advocacy service, made it easier to access support for changes in cognition. Whether this was letting participants know what was available, or might be helpful, or taking some of the load of navigating and completing the paperwork necessary when engaging with bureaucratic systems.

“They're an advocacy service. So, they put us in touch with somebody that works, I think, three days a week for them…she understands what was going on and just someone that has experience dealing with Centrelink, NDIS, doing applications, navigating that world of forms and phone calls and knowing who to call and what buttons to push to get things moving along, I guess.” (James, caregiver)

## 4 Discussion

This study explored the experiences of people with glioma and their caregivers regarding changes in cognition, cognitive assessment, and cognition-related support. This study found changes in cognition had considerable impacts for both people with glioma and their caregivers. However, ensuring healthcare professionals can identify and support these changes can be challenging. These findings have implications for both clinical practice and research.

### 4.1 Changes in cognition can be challenging to identify and support

This study supports what is already known about the ways changes in cognition can negatively impact people with glioma and their caregivers’ daily living, relationships, employment, leisure and quality of life (Schagen et al., 2014, Lucchiari et al., 2015, Olson et al., 2016). Despite these impacts, in the whirlwind early stages of diagnosis and treatment, cognitive concerns can become subordinate to survival. Although this intense experience occurs for a number of cancers, depending on stage, spread, and severity, the experience is potentially exacerbated in people with brain cancer due to the interrelation between the brain and people's sense of self (Eatough et al., 2012, Goebel and Mehdorn, 2019). This study showed that ensuring healthcare teams were aware of changes in cognition could be challenging due to inconsistent cognitive assessment, lack of cognitive insight on the part of the person with glioma, caregiver discomfort discussing cognition with the person they are supporting, and a lack of clarity over whether changes in cognition, behaviour, or mood were within the scope of medical care. Participants who accessed support for cognitive concerns found them helpful overall, however, participants indicated that it was difficult to find and access timely supports without self-advocacy or the support of an advocate, such as their Neuro–Oncology Care Coordinator.

### 4.2 Implications for clinical practice

This study highlighted a number of improvement opportunities for healthcare professionals working with people with glioma and their caregivers. First, although cognition may not appear to be a priority in the initial stages of diagnosis and treatment, cognitive changes can become incredibly impactful, therefore, timely cognitive assessment and appropriate supportive care referrals remain important (Ali et al., 2018, Pranckeviciene et al., 2017).

Routine cognitive assessment is important for people with glioma, however, it can be challenging to incorporate into busy cancer care clinics (Parsons and Dietrich, 2021, Morshed et al., 2021) even where a Neuro–Oncology coordinator is available - as was the case in this study. This study highlighted that people with glioma can experience multiple formal or informal cognitive assessment with various members of their treating team. Some people with glioma were administered similar screens or assessments on multiple occasions, which can result in inaccurate results due to practice effects (Beglinger et al., 2005, Duff et al., 2001, Cooley et al., 2015, Lee et al., 2022). These findings are in line with literature suggesting that in the absence of time and resources to complete gold-standard neuropsychological batteries, healthcare professionals are using briefer screens and/or subjective measures with people with glioma patients (Parsons and Dietrich, 2021). Healthcare professionals or health services interested in improving practices around identifying and supporting cognitive changes may benefit from identifying the healthcare professionals or services already conducting cognitive assessments with these patients and ensuring results can be shared with the wider treating team. Given Multi-Disciplinary Teams aim to enhance continuity of care for people with glioma (Oberg, 2019), the Multi-Disciplinary Team may have a role in streamlining the assessment and management process (Parsons and Dietrich, 2021). Furthermore, the inclusion of a Neuropsychologist in glioma Multi-Disciplinary Teams can ensure baseline and follow-up assessment and appropriate adjustments to treatment plans and referrals (Parsons and Dietrich, 2021, Oberg, 2019). However, it is also important to recognise that objective cognitive assessments used with people with cancer only capture objective changes to cognition, rather than the impacts these changes have on personality, relationships, and daily life (Olson et al., 2016, Lycke et al., 2019, Hutchinson et al., 2012). Objective cognitive assessment results should not be the sole indicator for referrals and should be completed in conjunction with subjective assessments as well as discussions with the person with glioma, caregiver, and healthcare professionals with the aim of contextualising symptom intrusiveness and ensuring people with glioma action and access supportive care referrals.

Communication between people with glioma, caregivers, and healthcare professionals was also identified in this study as a crucial component of identifying and supporting changes in cognition. It is important that healthcare professionals understand people with glioma and their caregivers may not clearly conceptualise their concerns as cognitive or as being explicitly related to thinking and memory. Rather, people may use less precise terms such as ‘wonky’, ‘woozy’, or ‘overwhelmed’. Being mindful of this may assist healthcare professionals in communicating with people with glioma and caregivers about changes in cognition. Likewise, healthcare professionals may improve communication about cognitive changes from people with glioma and caregivers by clearly communicating their role in management of all symptoms, including cognitive and psychosocial symptoms. It was noted in this study that not only do people with glioma sometimes lack insight into their cognition, but they may also be uncomfortable discussing these changes with a healthcare professional. Likewise, caregivers may feel uncomfortable discussing cognitive concerns in the presence of the person they are supporting. Therefore, providing opportunities for caregivers to directly communicate their observations with a healthcare professional may increase communication of changes or concerns. In this study, the Neuro–Oncology Care Coordinator was often identified as someone caregivers could contact directly and privately to share their concerns, indicating there is a role for Neuro–Oncology Care Coordinators, where available, as a point of contact for identifying cognitive decline via concerned caregivers.

Finally, people with glioma and their caregivers in this study who accessed resources or supportive care for changes in cognition found them beneficial. However, difficulties were reported regarding navigating and accessing these services without the support of a knowledgeable advocate. Healthcare professionals or health services interested in improving management of cognitive changes could benefit from identifying resources and local supportive care services that may be beneficial for people with glioma and caregivers, or identifying a Care Coordinator or Social Worker in the care team who can support people with glioma and caregivers to navigate these resources (Oberg, 2019, Willis et al., 2013). Development of structured referral pathways may also be helpful to ensure equitable access to resources and supportive care.

The role of Neuro–Oncology Coordinators in providing opportunities for direct communication about cognitive concerns, helping to navigate supportive care, and advocating for people with glioma and caregivers was ever-present in this study. The existing role of Neuro–Oncology Care Coordinators is to collaborate with the wider healthcare team, assisting people with glioma and caregivers to navigate health services, develop meaningful relationships with people with glioma and caregivers, and advocate for those in their care (Nixon and Morison, 2019, Bailey, 2015). Consequently, Neuro–Oncology Care Coordinators are ideally positioned to have role in coordinating timely screening, communicating results to the wider healthcare team, and implementing structured referrals. However, it is important to recognise that the Neuro–Oncology Care Coordinator role is a new and developing role embedded in a small number of (largely metropolitan) Australian healthcare services and often funded by charitable organisations (Nixon and Morison, 2019, Bailey, 2015).

### 4.3 Considerations for future research

This study has identified a number of areas that may benefit from further research. Firstly, this qualitative study identified the diverse way people with glioma and their caregivers can be impacted by cognitive changes in the months following diagnosis. Prospective longitudinal quantitative studies may build on this by identifying the prevalence of these impacts and the perceived benefits of utilised supportive care. Secondly, this study identified Neuro–Oncology Care Coordinators playing an integral and beneficial role for people with glioma and caregivers. Despite this, there is a paucity of data characterising the role of Neuro–Oncology Care Coordinators (Nixon and Morison, 2019, Bailey, 2015). Further research may consider clarifying the role of Neuro–Oncology Care Coordinators and the impact they have on patients, caregivers, healthcare professionals, and healthcare services. Thirdly, given the challenges for participants in identifying and navigating supportive care for changes in cognition. The field may benefit from systematically identifying or developing resources and supportive care services for people with glioma who are experiencing changes in cognition, and for their caregivers.

### 4.4 Strengths and limitations

Most participants in this study were caregivers. The inclusion of more people with glioma may have provided a greater diversity of views. However, recruiting people with glioma was challenging, partly due to the effects of the COVID-19 pandemic, and also reflects the small sample sizes seen in other studies with this patient group (Kirkman et al., 2023). Most participants were interviewed within six-months of diagnosis, therefore follow-up interviews or interviews with participants further from diagnosis may have yielded additional experiences. Some participants in this study had a family member present during the interviews, which may have limited the willingness of participants to be candid. However, no participants took the opportunity to review or amend their interview transcript. The sample was recruited from health services with a Neuro–Oncology Care Coordinator, a role that is charitably funded and only in place in a small number of health services in Australia (Nixon and Morison, 2019, Bailey, 2015). The proportion of people with glioma and their caregivers who have access to the expertise and support provided by this service is not known and appears to vary geographically. Therefore, the experiences reported in this study may not be representative of mainstream cancer provision. People with glioma and their caregivers who don't have a dedicated Neuro–Oncology Care Coordinator may report different experiences of cognitive assessment and navigating support.

## 5 Conclusion

This study found that in the early stages of diagnosis and treatment, the concerns of people with glioma and their caregivers about cognition can be overshadowed by the by nature of an experience which is intense and life-threatening. Despite this, changes in cognition were shown to have considerable impact on the experiences of people with glioma. The study data indicated that cognitive changes were challenging to identify and support due to inconsistent cognitive assessment, lack of cognitive insight, caregivers’ discomfort with discussions about cognition in the presence of the person they are supporting and challenges navigating support services. We suggest opportunities to identify changes in cognition and support people with glioma and their caregivers could include a Neuro–Oncology Care Coordinator-led, multidisciplinary approach to routine cognitive screening and structured referral pathways for people with glioma and caregivers, ensuring a direct line of communication between caregivers and healthcare professionals as well as consistent and equitable access to support.

## Funding sources

This pilot study is funded via a $100 000 grant awarded by the Sutton Family Brain Cancer Research Project Grant through the Mark Hughes Foundation. MC received a VC HDR scholarship from the University of Newcastle.

## CRediT authorship contribution statement

**Melissa A. Carlson:** Writing – review & editing, Writing – original draft, Project administration, Methodology, Investigation, Formal analysis, Conceptualization. **Elizabeth A. Fradgley:** Writing – review & editing, Resources, Methodology, Funding acquisition, Conceptualization. **Christine L. Paul:** Writing – review & editing, Supervision, Resources, Methodology, Formal analysis, Conceptualization.

## Declaration of competing interest

None.

## Appendix Supplementary materials

Image, application 1

Image, application 2

Image, application 3

## Footnotes

Supplementary material associated with this article can be found, in the online version, at doi:10.1016/j.ijnsa.2024.100179.
