# Supplementary material for: Understanding experiences of cognitive decline and cognitive assessment from the perspectives of people with glioma and their caregivers: A qualitative interview study
Source: Int J Nurs Stud Adv. 2024 Jan 17;6:100179. doi: 10.1016/j.ijnsa.2024.100179 (PMC11080318; doi:10.1016/j.ijnsa.2024.100179)
Supplement: Supplementary file 1 [file mmc1.docx]

Supplementary File 1: Demographic Characteristics

|  |  |
| --- | --- |
| Total | 18 |
| Participant Type |  |
| Person with glioma | 5 |
| Caregiver | 13 |
| Age |  |
| 30 to 39 | 4 |
| 40 to 49 | 5 |
| 50 to 59 | 1 |
| 60 to 69 | 5 |
| 70 to 79 | 3 |
| Gender |  |
| Female | 10 |
| Male | 8 |
| Other | 0 |
| Person they are supporting (support persons only) |  |
| Partner/Spouse | 10 |
| Parent | 3 |
| Relationship Status |  |
| Single, never married | 1 |
| Married/living with partner | 16 |
| Separated or divorced | 1 |
| Widowed | 0 |
| Aboriginal and/or Torres Strait Islander |  |
| Yes | 0 |
| No | 18 |
| Concession Card Holder^^[[1]](#footnote-1)^^ |  |
| Yes | 0 |
| No | 18 |
| Highest level of education |  |
| Year 10/School Certificate or lower | 4 |
| Year 12/Higher School certificate | 1 |
| Technical College or TAFE | 5 |
| Undergraduate | 5 |
| Postgraduate | 3 |

1. Concession cards are available in Australia for some people who are pensioners, carers, foster carers, receiving government financial support, low income earners and eligible veterans. The concession card entitles the holder to cheaper services and medicines. [↑](#footnote-ref-1)
