# Supplementary material for: Understanding experiences of cognitive decline and cognitive assessment from the perspectives of people with glioma and their caregivers: A qualitative interview study
Source: Int J Nurs Stud Adv. 2024 Jan 17;6:100179. doi: 10.1016/j.ijnsa.2024.100179 (PMC11080318; doi:10.1016/j.ijnsa.2024.100179)
Supplement: Supplementary file 2 [file mmc2.docx]

**
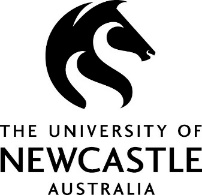
**

**Interview Guide**

**Interview Guide for the Research Project:**

**Lived experiences of cognition, cognitive assessment, and cognition-related supportive care for people with a primary brain cancer diagnosis diagnosis and their caregivers.**

Document Version 1; dated 15/07/2021

This document serves as a guide for conducting semi-structured telephone interview with participants of the above-named study.

The following questions may be used as a guide to elicit the interviewee’s ideas and opinions on topics of interest. However, the interviewer is able to follow trajectories in the conversation that may expand on the content of the guide when this is appropriate.

**Introduction**

Some people with a primary brain cancer can experience changes in their thinking and memory due to the tumour or its treatment. These changes can be temporary or more long lasting. We would like to know a little bit more about any experiences you/your [relationship to patient] have had of changes in thinking and memory.

**Experiences of changes in thinking and memory**

Could you tell me a little bit about you felt when you heard your/your [relationship to patient]’s cancer diagnosis?

Can you tell me about any changes you to your/their thinking and memory you may have noticed since the cancer diagnosis?

[If any] How have those changes impacted you?

What were you advised by the healthcare team about possible changes in thinking or memory that could happen as a result of the disease or the treatment?

[If advised] What was it like knowing that those kinds of changes could happen?

**Experiences of cognitive assessment**

Do you recall any of your health care team asking questions to test your/your [relationship to patient]’s thinking or memory?

*[If Yes] Tell me about which health care professionals raised it and when (eg after surgery); what sorts of questions did they ask?)*

*Prompts:*

- *How well do you think those questions captured any changes in memory or thinking, particularly the sorts of changes in thinking and memory that you described earlier?*
- *Was there anything in those questions which missed the point – e.g. didn’t take into account your gender, language, culture or identity?*
- *What kind of information or discussion (if any) did you have about what the answers to the questions meant about your/your [relationship to patient]’s thinking or memory?*

[If not described above] Could you describe any times you/they been offered any screens or tests that assess thinking and memory?

*(This could be a written or computerised test, or some questions you or a family member answer about thinking and memory)*

*Prompts –*

*-How well do you think those tests captured any changes in memory or thinking, particularly the sorts of changes in thinking and memory that you described earlier?*

*-Was there anything in those tests which missed the point – e.g. didn’t take into account your gender, language, culture or identity?*

*-What kind of information or discussion (if any) did you have about the results of the test?*

*-*

**Experiences of support for thinking and memory**

Can you tell me about any times you/they have been offered any support in relation to changes in thinking and memory?

*(For example, medication, therapy, brain training exercises, physical exercises, some support in the home, strategies to help you/them remember things better).*

*How much did the support help?*

What would you have liked to have been done differently in relation to thinking and memory?

What would be your advice to other people with brain cancer/caring for someone with brain cancer who have concerns about thinking and memory?
