# Supplementary material for: Understanding experiences of cognitive decline and cognitive assessment from the perspectives of people with glioma and their caregivers: A qualitative interview study
Source: Int J Nurs Stud Adv. 2024 Jan 17;6:100179. doi: 10.1016/j.ijnsa.2024.100179 (PMC11080318; doi:10.1016/j.ijnsa.2024.100179)
Supplement: Supplementary file 3 [file mmc3.docx]

**Supplementary File 3. Detailed Methods**

Design

Cross-sectional qualitative semi-structured qualitative telephone interviews were completed and analysed using reflexive thematic analysis [30]. The project was approved by Hunter New England Human Ethics Committee (2019/ETH11694).

Study Setting and Participants

Eligible participants were: eighteen years of age or older; had received a glioma diagnosis (herein referred to as people with glioma), or were supporting someone with a confirmed glioma diagnosis (herein referred to as caregivers); participated in a pilot study of the Audio Recorded Cognitive Screen [31] and attended at least two appointments within a six-month time frame at one of the study sites. This timeframe ensured there was opportunity for both cognitive assessment and discussion of supportive care. Caregivers who had completed an online survey as part of a pilot study and indicated they could be contacted about further research were first invited to participate in this study; then the person they were caring for was invited to participate. This approach was chosen to be sensitive to the challenges of treatment and recovery for people with glioma. All participants were given time to review an information statement outlining the reason for conducting the research prior to consent.

Procedure and Measurement

Telephone interviews were conducted between 13th September 2021 and 8th March 2022 by a PhD Candidate (MC) with qualifications and skills in social science methods and experience in qualitative research with people with a cancer diagnosis and their caregivers. The interviewer (MC) is currently conducting PhD research exploring cognition with people with glioma and their caregivers and has had previous and ongoing contact with participants through another study piloting cognitive assessments [25]. A semi-structured interview guide (See Supplementary File 2) was developed exploring: experiences, observations, and feelings about perceived or objectively measured changes in thinking and memory (or absence thereof); experiences of thinking and memory assessment, and; experiences of met and unmet supportive care needs related to changes in thinking and memory. Some participants chose to have a caregiver with them while completing the interview. No participants accepted the offer to review their interview. As per the ethically-approved protocol, at the end of each interview, the interviewer asked the participants if they were experiencing any emotional discomfort. Where appropriate the interviewer offered participants the telephone number for the Cancer Council Information and Support Service (13 11 20) or to have their Neuro-Oncology Care Coordinator call them to follow up. Although participants were experiencing many challenges, no participant reported experiencing additional emotional discomfort as a result of the interview. Those who raised queries or concerns expressed a preference and capacity to follow-up directly with the Neuro-Oncology Care Coordinator themselves.

The interviews were between two and 17 months from diagnosis, were between 17 and 56 minutes in duration, audio-recorded on a tablet, transcribed by a professional transcription service, de-identified and imported into NVivo 12 software. Reflexive field notes were used to record team observations and reflections. Authors (MC, CP, EF) met throughout recruitment and data collection to reflect on the interviews and field notes. During this process it was agreed that the achieved sample of participants provided rich breadth and depth for analysis (as favoured over data saturation in thematic analysis [30]). A subset of these data regarding participants’ experiences of specific aspects of the cognitive assessment were analysed separately and reported elsewhere [32].

Analysis

Reflexive thematic analysis [30] was chosen to allow an in-depth, inductive and interpretive approach which can be used to identify patterns within the data, and is theoretically flexible [30]. This theoretical flexibility allowed the research to be underpinned by critical realism, which can both centre participants’ voices while also situating those voices within the wider context of health systems and practices [30, 33]. The transcripts were coded by one researcher (MC) in a collaborative and iterative process with three members the research team (MC, CP, EF). Analysis began following the first interview when the first author met with the other authors to share reflections on the first interview. Throughout the data collection, analysis, and writing period, the first author would share summaries and reflections from their interviews, field notes, and analysis for discussion during regular meetings with the other authors. The first author read and re-read the initial three transcripts before hand-coding these transcripts with codes and definitions. Following this process, initial codes were discussed with the research team, interrogated, and further developed. Following this, the first author re-coded these transcripts in NVivo. This collaborative process continued as the first author coded transcripts alongside data collection with regular discussion of new or changing codes and grouping codes into categories. Upon completion of coding and categorisation, the first author explored patterns in the data, developing a map of potential themes for discussion. The themes were then collaboratively refined by the authors before the first author made a theme matrix which included a list of themes, a summary of the theme, and illustrative quotes. The authors met to refine these themes and ensure the data were captured by these themes. The themes were discussed amongst the research team and further refined through the writing process [34]. The themes presented are the outcome of this process.
